# Celebrity Couples as Business Families: A Social Network Perspective

**DOI:** 10.1177/08944865211050348

**Published:** 2021-10-18

**Authors:** Yasaman Gorji, Michael Carney, Rajshree Prakash

**Affiliations:** 1 Concordia University, Montreal, Quebec, Canada

**Keywords:** business families, celebrity capital, project-based industries, social networks, nexus of contracts

## Abstract

We depict Hollywood celebrity couples as business families who participate in the project-based movie production industry, which is a temporary and disaggregated form of organization where skilled individuals are linked to one another through contractual and social relationships. Appearing in Hollywood movies generates celebrity capital, which can be converted into economic capital through involvement in endorsements and other rent-generating activities. Finding projects is facilitated by membership in high-quality social networks, and we consider celebrity marriage as a means of merging two individuals’ social networks, which can be mutually beneficial for both parties. We develop and test three hypotheses about the quality of social networks prior to and after marriage and analyze their impact upon celebrities’ postmarriage career performance. We contribute to the family business literature by exploring hybridized and adaptive forms of business family in contemporary project industries, which has the potential to enlarge family business scholars’ research horizons.

## Introduction

Much of the recent family business literature views business families from a “one family-one firm” perspective and focuses on how family resources or significant family events influence firm outcomes (Brinkerink et al., 2020; Combs et al., 2020). However, the “one family-one firm” approach to business families underplays the worldwide prevalence of serial entrepreneurs and business families with stakes in multiple businesses (Masulis et al., 2011). Moreover, business families hold significant wealth beyond the firm in other types of financial and nonfinancial assets (Carney & Nason, 2018). Accordingly, our study of celebrity couples in business expands our understanding of the scope of business family involvement in modern global industries. While marriage can be instrumental in creating and perpetuating dynastic business families (Landes, 2008; Lisle-Williams, 1984), our depiction of marriage as establishing a family in business is generally absent from family business research.

Theoretically, we portray celebrity couples as a particular form of a business family. Celebrity is a specific form of social capital co-constituted by the achievements of an individual, mediated through social and legacy media to audiences in the general public (Driessens, 2013). Celebrity capital can be amplified and converted into other resources, such as economic and political capital (Driessens, 2013). For example, through endorsements and the commercial use of image rights, celebrity capital can be converted into reputational capital to promote the success of new business ventures (Hunter et al., 2009). Celebrity capital can also be amplified through intermarriage with another celebrity (Parmentier, 2011). The marriage between Hollywood actors Brad Pitt and Angelina Jolie in 2005 set a new standard for merging the identities of two individuals (Brangelina), establishing “a new level of celebrity” (Díaz, 2020, p. 275). Celebrity is a substantial global industry where celebrity couples such as Kanye West and Kim Kardashian are reported to have accumulated a combined net worth of some $3.5 billion (Western, 2020).

We address the phenomena of celebrity business families through the lens of a social network perspective because success in many areas of a contemporary business depends on social networks comprising both strong and weak ties (Burt, 2000; Coleman, 1998). Granovetter (1973) established that access to valuable information could be better achieved through weak ties, especially when finding work (Granovetter, 2018). Marriage represents a strong tie that can provide entry points to networks of well-connected people (Stewart, 2003). How ties of variable strength are combined in networks surrounding business families is underexplored in the literature. However, recent research suggests that variability in social networks’ openness is associated with hybridized family firms (Burt et al., 2021).

Social networks are instrumental in industries populated by project-based organizations, an organizational form comprising temporary structures for the performance of discrete, project-based tasks (Lundin et al., 2015; Sydow et al., 2004). Many industries are shifting toward project-based organization, characterized by freelancing, short-term, contractual assignments. These industries include professional services such as architecture and design (Teece, 2003); cultural industries such as event management, theatre, film and television production (DeFillippi, 2015); and high technology such as software, video games, computer hardware, multimedia (Hobday, 2000). In these industries, individuals move serially between employers and are selected based upon reputation, prior achievements, as indicated by technical and market performance. The research setting for this article is celebrity couples in Hollywood’s film production industry, considered the prototype of a project organization industry (Jones & Walsh, 1997).

However, celebrity can be fleeting, and Hollywood artists must seek to maintain and extend their celebrity by finding new roles in film projects, which membership in social networks facilitates (Faulkner & Anderson, 1987; Lutter, 2015). Accordingly, we develop and test three hypotheses about the size and quality of celebrity couples’ social networks and their effect on finding new projects before and after marriage in the context of the U.S. film production industry. We employ data from the International Movie Database (IMDb), which contains career data points on 17,000 couples who have produced, directed, or cast in U.S. feature films. We select a sample of 1,168 married couples active in Hollywood between 1970 and 2010. This study seeks to understand better how celebrity marriages represent the pooling and borrowing of spouses’ social ties derived from their networks, with potentially positive benefits for both (Burt, 2000).

A primary contribution of this article is to extend the family business literature by demonstrating that business families can participate in multiple business lines without owning a firm in the traditional sense of the word. As carriers of an inalienable strategic asset, celebrity capital, we show that celebrity couples participate in “an established commercial enterprise made up of a highly developed institutionalized structure of linked professions and sub-industries” (Driessens, 2013, p. 547). Theoretically, we draw upon Gedajlovic and Carney’s (2010) transaction costs theory of the family firm, which depicts the firm as a nexus of contracts governing intangible strategic assets such as social capital, reputation, and tacit knowledge. While we situate our empirical study in the Hollywood film production industry, we propose that the business families as a nexus of contracts apply more generally to industries populated by project organizations. Other types of intangible capital, such as human and social capital, can be mobilized and amplified through the pooling of married couple’s social networks. By enlarging the scope of business family research beyond the traditional family firm, we identify research opportunities for family business scholars to examine the functioning and performance of business families in modern sectors of the economy. Recognition of married couples as families in business enlarges the range of organizational forms where strategic outcomes can be better understood when examined through family logic.

## Theory and Hypotheses

### Business Families as a Nexus of Contracts

Previous literature identifies several streams of research on marriage and the family firm. One stream of research shows how marriage creates mutual and productive obligations that underpin spousal entrepreneurial teams (Bird & Zellweger, 2018; Fitzgerald & Muske, 2002). Other studies find that marriage can reinvigorate the firm by infusing new nonfamily talent (Mehrotra et al., 2013). Similarly, the marriage of a family business member to a partner from a prominent business or political family can generate significant increases in firm value (Bunkanwanicha et al., 2013). Recent reviews have advocated greater use of family science in family business studies (Combs et al., 2020; Jaskiewicz et al., 2017), with the suggestion that business family attributes such as family transitions (e.g., marriage) can influence firm outcomes. Much of this literature is about how marriage can beneficially contribute to family firm performance. The distinguishing feature of our business families is that as celebrity couples rarely own firms, instead their economic goal is to generate economic rents from roles in project organization production.

As an economic organization that mobilizes and configures resources toward commercial ends, celebrity couples participate in the film production industry as carriers of inalienable commodities, talent, and celebrity. No individual “owns” the temporary project organization. Instead, participants carefully specify the contributions, property rights, risks, and returns of a project in a series of contractual relationships. In this respect, project organizations resemble Jensen and Meckling’s (1976) characterization of the firm as a legal fiction, which serves as a nexus of contracting relationships among individuals in various professional capacities.

The notion of the firm as a nexus of contracts was amenable to transaction costs theorists (Williamson, 1990), who argued that efficient governance of specific assets calls for different transaction modes, including classic and relational contracting. However, transaction costs theorists observed that certain types of assets were nontradable in factor markets, meaning that intangible assets, such as trustworthiness and reputation, were sticky to the firm that created them (Teece, 1986). In their transactions costs theory of the family firm, Gedajlovic and Carney (2010) proposed that family firms represent an efficient governance mechanism for developing and extracting value from generic nontradable assets (GNTAs). This category of assets is specific to the firm but generic in their business applications, which they describe as social capital, reputation, and tacit knowledge. For example, an entrepreneur with a reputation for integrity may enter several lines of business (Masulis et al., 2011). Because GNTAs are nontradable they must be leveraged by the individual or firm to which they are attached.

We reason that celebrity capital is a form of GNTA: Celebrity capital is personalized and sticky or inalienable to an individual. Celebrity capital is convertible and enables individuals to participate in elite social networks and attract economically valuable partners. Celebrity capital is generic in that it can be applied to a range of activities, such as philanthropy or politics (e.g., Trump, Schwarzenegger). Celebrity capital is particularly valuable in business through product endorsements or participating in new entrepreneurial teams to establish new businesses (Hunter et al., 2009). For example, multi-Oscar-award winner director Steven Spielberg cofounded DreamWorks with David Geffen and Jeffrey Katzenberg. In this regard, we suggest celebrity as a form of GNTA because celebrity couples enjoy advantages in developing and appropriating value from their celebrity.

However, to do so celebrity couples do not necessarily own a particular firm, although they are often the public face of cofounders of entrepreneurial ventures. Rather, they are contractually linked to a broader group of subprofessions (Driessens, 2013) and experts (Teece, 2003). Perhaps the most essential contractual relationship for Hollywood celebrities is the talent agent, who finds roles and negotiates terms for their clients on a commission basis. Second, many celebrities contract for personal managers who guide their clients’ careers in selecting roles (Roussel, 2017; Zelenski, 2002). Moreover, celebrities also rely upon a retinue of contracted experts, such as stylists, voice and fitness coaches, publicists, social media strategists, personal assistants, lawyers, accountants, and investment advisers (W. T. Bielby & Bielby, 1999; Zafirau, 2008). However, the functional structure of the firm as a nexus of contracts depends crucially upon the size and quality of a celebrity’s social networks. In other words, in highly differentiated and disaggregated industries, the firm as a network of contracts is also about network embedding (Burt et al., 2021).

### Social Networks and the Firm as a Nexus of Contracts

In project-based labor markets, social networks enable access to valuable information, reduce search costs, foster careers by helping individuals acquire assignments, and establish opportunities for future collaborations with others who provide social and emotional support (Lutter, 2015). Briefly, the quality of a celebrity’s social networks derives from connections between people by which they can directly or indirectly assist one another in terms of career advancement. This is the sense in which social networks will be used throughout this analysis to shed light on how social networks can help individuals identify avenues for career advancement.

Social networks differ according to the individuals within the network and the structure of their relationships with each other. Hence, social networks can be strong and closed (Coleman, 1988) or weak and loose (Granovetter, 1973). These differences generate different values. Closed networks, or cliques, provide members with fewer informational advantages as the number of interconnections is high, but the ties are highly redundant (Burt, 2000). Still, closed networks can foster a greater sense of emotional support and trust among members, increasing their willingness to collaborate in future projects (Lutter, 2015). Alternatively, Granovetter (1973) argues that weak social networks provide better information and have better career-enhancing value. The individuals who comprise weak social networks are loosely associated and can often bridge what Burt (1992) describes as structural holes. This may provide these individuals better access to information that is not readily available in a closed network.

We develop and test three hypotheses concerning this general premise; the first hypothesis predicts that marriage benefits spouses by allowing the receiving spouse to capitalize on the supporting spouse’s social ties. This baseline hypothesis predicts a mutually beneficial outcome in which both spouses increase the annual number of films (projects) compared with the annual rate of their premarriage projects. The second hypothesis predicts career elevation to executive roles in the industry whereby marriage benefits spouses by enabling the receiving spouse to capitalize on supporter’s accumulated social ties to ascend the industry’s career hierarchy through recruitment into executive roles. The third hypothesis is gender-specific, predicting that marriage amplifies benefits for women by enabling the receiving spouse to capitalize on her spouses’ accumulated social ties such that it curbs gender bias to facilitate women’s participation in executive roles (Bevelander & Page, 2011; Burt, 1998).

### Marriage, Social Network Characteristics, and Career Outcomes

Marriage is a strong social tie that represents an opportunity for the unification of two previously independent social networks with potentially reciprocal benefits. These benefits are dependent on the size and quality of the participants’ network. Below we provide a concise summary of the size and quality of social networks. Regarding network size, some people have extensive social networks as they are connected to many others, in terms of network theory, this is referred to as degree centrality (Freeman et al., 1991). For example, novice actress Michelle Pfeiffer with only two movie credits, had a low degree centrality when she married the slightly more established actor Peter Horton in 1981. Subsequently, both went on to more successful careers with Pfeiffer being nominated for three Oscar awards. Thus, marriage may pool spouses social networks; and supporting spouses can share their respective ties and provide multiple new links to access information and resources (Bellow, 2004). Individuals with fewer ties are relatively disadvantaged (Sparrowe et al., 2001). However, in addition to network size, other quality characteristics can influence the value of a social network.

We consider two salient quality characteristics of social networks: an individual’s status in a network and, second, their capacity to bridge unconnected social networks. Status is “the hierarchical position of an actor within a social system” (Jensen & Kim, 2015, p. 1). Hierarchical social networks differ from nonhierarchical social networks in that much information flows through one or more central individuals, within a hierarchical network. Individuals with connections to other influential high-status network members are more central and have a high Eigen centrality (Bonacich, 2007; Borgatti, 2005). For example, actress Trish Van Devere played roles in just three TV episodes when she married the higher status and Oscar award-winning actor, director, and producer George C. Scott in 1972. Subsequently, she appeared in many of Scott’s movies, especially when he was in the leading role. Later on she was cast independently, appearing in higher quality films and TV series. Note that network status is not equivalent to career status where an artist accumulates numerous project roles. Rather network status relates to the quality of other artists to whom the focal person is connected. For example, the renowned auteur director Terrence Malick directed only three films between 1973 and 1997 but is among the most well-connected and high status directors in Hollywood.

The second aspect of quality concerns the capacity to bridge social networks that are otherwise unconnected. Bridging is the capacity to forge ties with actors in unrelated networks or bridging structural holes. Information tends to flow fully and freely within closed networks, providing certain benefits, but information circulating in closed networks is less valuable because everybody has it. In contrast, information flows much more sporadically across structural holes and as such, information that flows in these channels tends to be more valuable and nonredundant. Considered to be the most important source of value are individuals whose structural position is such that they can bridge structural holes between two unconnected social networks (Burt, 2000). Therefore, individuals who can bridge structural holes can better broker information as bridging enables information filtering, creating advantages, and power within and between networks.

Burt (1998) proposes that an individual situated, in a hierarchical social network, that is, someone who is connected to other higher status persons, has the capacity to “lend” social capital to lower status or peripheral individuals so that they can benefit from the high-status individual’s position. Burt (1998) finds that women and younger men tend to be lower status members of organizations and networks. For example, renowned multi-award winner director Brian De Palma, in his first short movie directed Robert De Niro. Subsequently, De Palma went on to direct a diverse range of films with well-known actors such as John Travolta, Al Pacino, Kevin Costner, Sean Connery, and Andy Garcia, which linked him to different cliques in the movie industry. De Palma’s future wife, Nancy Allen was a peripheral figure in the industry, appearing mainly in TV commercials, DePalma cast Allen in leading roles in a series of high-profile movies, including *Carrie*, *Dressed to Kill*, subsequently she garnered mainstream fame playing in Paul Verhoeven’s *Robocop* sequels. In this relationship DePalma is “lending” his social capital to promote his spouse’s career. Here we note that by borrowing social capital from a higher status individual does not represent a reciprocal relationship, at least not in the initial stages of the marriage.

In the context of project organizations, each of these social network characteristics, degree centrality, status, and the capacity for bridging structural holes are potentially valuable mechanisms to a receiving spouse seeking career advancement in their field. Accordingly, we evaluate the size of each spouse’s social network, using degree centrality. For quality, we used status (Eigen centrality) and the capacity for bridging structural holes (Burt’s constraint). Therefore, we hypothesize that

**Hypothesis 1a:** The size (Degree centrality) of a supporting spouse’s network will be positively related to the postmarriage career outcome of the receiving spouse.
**Hypothesis 1b:** The quality (Eigen centrality and Burt’s constraint) of a supporting spouse’s network will be positively related to the postmarriage career outcome of the receiving spouse.

### Marriage and Promotion in the Career Hierarchy

Like formal organizational structures, single project organization may also display elements of hierarchy (Starkey et al., 2000). In these structures certain positions possess greater authority to coordinate projects, allocate resources, and exercise executive control over subordinates’ activities. In the Hollywood movie production industry, directors and producers possess such authority over actors and technical crew. While producers are usually appointed by production financiers, high-profile actors are often interested in exercising control over the way they are depicted in the movie. Indeed, high-profile actors frequently coinvest in the movies in which they appear to secure executive producer positions.

The rise of intraprofession marriage (Compton & Pollak, 2007) indicates a need for better understanding about how gender affects the networks and career outcomes of spouses (Sorenson & Dahl, 2016). In particular, stereotypes about female executives are remarkably consistent across cultures and have remained relatively stable over time. These stereotypes cast women as more gentle, caring, nurturing, and communal than men. On the other hand, men are labeled as more assertive and competitive, or agentic (Carli & Eagly, 1999; Williams & Best, 1990). Given these stereotypes, women face two challenges concerning managerial positions. First, many people view women as lacking the requisite characteristics to become good managers. Such characteristics are often considered to be masculine qualities. Both men and women exhibit a preference for homophilous network choices (Mouw, 2003). Homophily is the tendency for individuals to form groups with others who share common characteristics such as gender, race, socioeconomic status (McPherson et al., 2001). However, the effects of homophilous network preferences on career outcomes differ for males and females. White middle-class men tend to have more powerful and better quality social networks than do women. Women, who can be excluded from many male social networks, also exhibit homophily and associate more closely with other women (Aldrich et al., 1989). Because women tend to have lower status jobs, the resulting social networks that they form tend to be less potent than those of men (Groysberg, 2010; Ibarra, 1992). Given the importance of information that flows through a social network for career advancement to senior roles, women often do not have access to the same high-quality information available to men, which limits their career opportunities (Lutter, 2015). To compensate for their social network and social capital disadvantage, women often seek a male mentor and borrow social capital from that sponsor to advance in their careers (Burt, 1998). Yet other research finds that women often have difficulty finding male sponsors for myriad reasons (Groysberg, 2010; Lutter, 2015).

However, with this hypothesis, we reason that some of the detrimental career effects of female stereotyping and social network formation may be diminished by marriage, especially in project-based career environments, which differ from traditional careers. Indeed, the pooling of social capital within-profession marriage in project-based industries can be reciprocally beneficial; it is a dynastic strategy producing synergistic effects for both spouses. First, due to the strength of the marriage tie, we propose that both spouses will be willing to lend one another social ties through mutual sponsorship. Second, due to the importance of social ties in securing projects, both spouses will be interested in maximizing the size and quality of social networks, and both spouses have incentives to look out for each other’s interests. Given the importance of social networks in project-based industries (Jones & Walsh, 1997; Lutter, 2015), we expect that spouses in the same industry will seek to leverage their collective capital to improve both spouse’s positions. Therefore, we hypothesize that

**Hypothesis 2:** Following marriage, a supporter’s social capital can provide greater access to receiving spouse to achieve higher level production management roles.

With our previous hypotheses, we have argued that the effects of marriage on social capital and social network dynamics concerning sponsorship, homophily, and gender stereotyping can improve both partners’ career performance. Reciprocal sponsorship may be especially beneficial to women who typically have difficulty finding high-status male sponsors. With our third hypothesis, we provide a gender-specific rationale suggesting that marriage may accentuate females’ career performance by breaking the glass ceiling to ascend from operational or performing roles to executive positions.

Women face considerable barriers to employment and career advancement in the U.S. film industry due to the risk profile associated with big-budget blockbuster projects. Project risk inhered in the industry’s reliance on social networks, social capital, and the associated elements of reputation and status effectively bar women from achieving senior project management positions (D. D. Bielby & Bielby, 2002; Grugulis & Stoyanova, 2012; Lincoln & Allen, 2004). Due to the presence of homophily, women have less access to influential social networks with valuable social capital and therefore are at a distinct disadvantage in Hollywood (Lutter, 2015). Added to this situation are gender stereotypes and the blockbuster strategy-driven risk inherent in most filmmaking, which further prohibits women from securing management roles (Faulkner & Anderson, 1987; Lutter, 2015). The combination of these effects and factors has resulted in an environment in which women are absent from senior management positions, are paid less, and exit the industry with a higher frequency than men (D. D. Bielby, 2009; D. D. Bielby & Bielby, 2002; Lincoln & Allen, 2004).

The apparent prevalence of male preferment may arise because Hollywood’s celebrated code of cultural norms conceals taken-for-granted preferences. Based on recent disclosures, particularly about prominent film producers, these norms can also result in sexual harassment (Chen, 2018). The problem may be accentuated for women seeking managerial roles in Hollywood, or who improve their status. Both threaten gender stereotypes that pervade the industry, and to reinforce male-dominated social networks, together, these factors can make Hollywood a closed shop for women. Furthermore, while there is a growing social consensus that women make good managers, gender stereotypes preclude women from senior management roles (Carli & Eagly, 1999). Women are discouraged from seeking such positions (Eagly & Mladonic, 1994). The subjective nature of Hollywood’s hiring process mostly favors men because of homophily and ungrounded belief that women are riskier hires (D. D. Bielby, 2009).

Allowing for the unfriendly environment facing women in Hollywood, it becomes evident why they might be more discerning about the connections that they form, including the individuals from whom they borrow social ties. In combination with the importance and value of family social ties for all family members (Gorji et al., 2019), these factors appear likely to result in an increased reliance on social ties borrowed from family members or spouses. As such, we expect spouses in Hollywood to lend and borrow social ties from one another. Furthermore, given Hollywood’s male-centric environment, it could be advantageous for female spouses to place greater trust in their spouses as a source of sponsorship. Accordingly, women will tend to benefit more from borrowed social capital, given their supposedly lower status in most organizations and networks (Burt, 1998). Given the desirability of a trusted sponsor, we expect that a female spouse will borrow social ties from her spouse, providing a greater probability of career advancement than unmarried women. Thus, we hypothesize that

**Hypothesis 3:** Following marriage, women benefit more than men from borrowed spousal social capital to achieve higher level production management roles.

## Data and Method

### Data

We take our data for this analysis from archival Hollywood information contained in IMDb, an online database that provides relevant career information about Hollywood films, including cast, crew, directors, producers, editors, and all associated individuals who receive a production credit. We selected the IMDb and Hollywood as an industry for two principal reasons. First, the IMDb contains sufficient information about all associated individuals and their marriage(s) under the relevant life details, including date of marriage, divorce, and death. With these data, we can determine the timing and duration of each marriage. This information is essential to evaluate the effect of marriage on their subsequent career performance. We use repeated event analysis history to model the sometimes-complicated marriage and divorce history of sampled individuals. Second, scholars consider Hollywood to be an exemplar of a project-based industry with a boundaryless career structure (Lutter, 2015). The requisite data are necessary to evaluate the temporal trajectory of an industry participants’ career performance with objective performance measures.

### Sample

Our analysis uses a sample size of 1,168 married couples who were active in Hollywood between 1970 and 2010. Married couples considered in this analysis had credits in a specified number of Hollywood films. Spouses are included in this study if they have a minimum of three film credits before marriage and must continue working in the industry for at least 3 years following marriage. The spouses had to be active for at least 5 years in the U.S. film industry before their marriage, and at least one spouse had to continue working in Hollywood for at least 3 years after the marriage date. Furthermore, we selected only individuals who had worked as part of the core crew on a Hollywood film for this analysis. The core crew consists of a movie’s producers, directors, actors, actresses, writers, editors, cinematographers, production designers, and soundtrack composers (Cattani & Ferriani, 2008; Goldman, 1983). We consider producers and directors to be hierarchically superior in the career structure due to their influence over decision-making in production. Given Hollywood’s boundaryless career structure and the importance for the U.S. film industry, we reason that only core crew members would possess the necessary social ties to lend to a spouse to improve his or her career trajectory.

The sample does not comprise individuals who own a firm. Instead, ours is a sample that permits us to observe social network dynamics of marriage within a project-based industry. The business family literature is sometimes concerned with marriages as a means of intergenerational wealth preservation and the perpetuation of elite roles in society (Bika & Frazer, 2020; Landes, 2008). However, as noted above, business families are typically reluctant to disclose their practice; thus, scholars are blind to how marriage can promote positive outcomes. Hence, readers should view our findings as an opportunity to gain insight on how elite social networks function to advance or reproduce favorable results for their members (DiMaggio & Garip, 2012).

### Dependent Variables

We use two dependent variables to measure an individual’s career performance. The first is a *simple quantit*ative measure: the absolute number of projects defined as core crew roles in movie production. Since this measure is a count variable, we analyze these data with negative binomial regression (Gardner et al., 1995; Ver Hoef & Boveng, 2007). To determine the effect of marriage on career performance, we collect data for 5 years before and 3 years after the marriage. The timeframe examined after marriage was intentionally made shorter to isolate the effect of marriage on each spouse’s career performance.

The second dependent variable is a categorical measure that examines any positive or negative change in roles concerning a productions’ hierarchy (i.e., moving up toward producer or directing roles, or demoted from such positions to acting or writing roles). We categorize role changes in three ways: *Promote*: upward movement from acting to producing/directing roles; *Demote*: downward movement from producing/directing roles to acting and other core crew roles; *No change*: represents remaining in the same role category in the following 3 years after marriage. Because this dependent variable is categorical, we employ multinomial regression with more than two categories (Long, 1997).

### Independent Variables

Our independent variables are the size and quality of a supporting spouse’s social network. For size, we measure each spouse’s degree centrality (i.e., the number of ties or connections a node has within the movie core crew). For quality, we use two measures: Eigen centrality and Burt’s Constraint. Eigen centrality tracks hierarchical status, which indicates the ability to influence others in a network. Burt’s constraint describes the extent to which a person’s network is concentrated in redundant contacts (Burt, 1992). A low constraint indicates an ability to bridge structural holes. A high constraint occurs if contacts are directly connected (dense network) or indirectly connected via a central connection (hierarchical network).

### Control Variables

Reflecting the accumulated literature on social capital, career, and family business, we control for several variables to rule out alternative explanations of postmarriage career effects. First, we control for age at the time of marriage since age typically has a positive effect on men and negatively impacts women (De Pater et al., 2014). This effect is due to the diminishing audience appeal of older women in lead roles (Treme & Craig, 2013). Given the discrimination encountered by women in the industry, we control for gender. For Hypotheses 1 and 2, gender serves as a control variable and as a moderating variable in the third hypothesis. Industry experience, measured in years, is our third control variable at the time of marriage because individuals typically accumulate industry-specific experience, which will extend their networks (Jones, 1996). Due to the relatively high correlation between the number of projects before and after marriage (*r* = .566, *p* < .001), we decided to use previous projects as a control variable. Our second categorical dependent variable (change of role) also has a high correlation with a spouse’s role before marriage (*r* = .646, *p* < .001); hence, we also control for receiver’s role type before marriage. We also control for the type of marriage termination based on our collected data. Typical forms of marriage termination include divorce, death, and separation. The natural death of the spouse as a reason for marriage termination might present a more supportive relationship compared with divorce or separation. Finally, recipients of Oscar awards enjoy higher visibility, typically leading to increased numbers of projects (Jensen & Kim, 2015). Thus, we control for Oscar awards before marriage.

### Analysis

For our analysis we use Stata 15 software to perform negative binomial regression, multinomial regression, *t* tests, and a correlation table. Our dependent variable, “number of projects” *or movie roles*, is a count variable. If the outcomes are discrete count variables, then Poisson regression or negative binomial regression can be used (Block et al., 2013; Verbeek, 2004). We use negative binomial regressions whenever the sample variance of the dependent variable exceeds its sample mean (i.e., overdispersion), while Poisson distribution assumes that the mean and variance are the same. Due to this difference, we selected a negative binomial regression over Poisson due to its flexibility for data that shows overdispersion. To measure network centrality, we use R studio Version 1.0.143. We applied the “Constraint” command in the “iGraph” library to calculate the egocentric-based Burt’s constraint. To measure degree and Eigen centrality, we used social network analysis package in R. Table 1 presents the correlations among the variables, the means, and standard deviation.

**Table 1.** Descriptive Statistics and Correlations.

Variables	Bivariate correlations																
	Mean	*SD*	(1)	(2)	(3)	(4)	(5)	(6)	(7)	(8)	(9)	(10)	(11)	(12)	(13)	(14)	(15)
(1) Receiver’s type of role BM	1.65	0.890	1.00														
(2) Receiver’s role after marriage AM	1.86	0.890	−.128^**^	1.00													
(3) Receiver’s change of role AM	1.19	0.679	−.646^**^	.702^**^	1.00												
(4) Receiver’s experience BM	8.54	9.465	.309^**^	−.080^**^	−.186^**^	1.00											
(5) Receiver’s award BM	0.03	0.162	.108^**^	.103^**^	0.05	.125^**^	1.00										
(6) Receiver’s age at marriage	34.7	10.437	0.163^**^	0.238^*^	−.073^*^	.512^**^	.069^*^	1.00									
(7) Type of marriage termination	1.21	0.408	0.05	−.160^**^	−.126^**^	.376^**^	−0.02	.462^**^	1.00								
(8) Receiver’s count of projects (AM)	0.55	0.772	.255^**^	.092^**^	−.088^**^	.137^**^	.151^**^	−.084^**^	−0.03	1.00							
(9) Receiver’s count of projects BM	0.47	0.659	.375**	.358^**^	−.232^**^	.255^**^	.110^**^	.046	−.025	.565^**^	1.00						
(10) Receiver’s degree BM	12.94	34.308	.198^**^	.101^**^	−0.05	.159^**^	.155^**^	−.029	−0.03	.331^**^	.502^*^	1.00					
(11) Receiver’s Eigen centrality BM	0.002	0.038	0.02	0.01	−0.01	0.06	0.00	−.108^**^	−0.02	.057^*^	−.109^*^	.231^**^	1.00				
(12) Receiver’s constraint BM	0.015	0.052	.121^**^	0.02	−0.03	.106^**^	.158^**^	.032	−0.04	.341^**^	.407^**^	.254^**^	0.02	1.00			
(13) Supporter’s degree BM	11.38	32.177	.083^**^	.081^**^	0.02	0.04	0.04	−.114^**^	−0.03	.070^*^	.184^**^	.184^**^	0.05	0.04	1.00		
(14) Supporter’s Eigen centrality BM	0.001	0.026	0.02	0.01	−0.01	0.03	−0.01	−.071^*^	−0.01	0.03	.075^**^	.075^**^	.076^**^	0.02	.115^**^	1.00	
(15) Supporter’s constraint BM	0.013	0.049	0.03	0.02	0.00	0.02	0.00	−.091^**^	−0.02	0.02	0.04	0.04	0.01	.081^**^	.262^**^	0.03	1.00

*Note*. BM = before marriage. AM = after marriage.

* *p* < .1. **p < .05. ***p < .01.

## Results

Our findings offer strong support for some of the hypotheses while also corroborating elements in the academic literature pertaining to borrowed social capital and gender bias. The results also offer novel insights about the value of marriage for men and women and the value of their social ties in the Hollywood context. *Hypothesis 1* states that the size (*Degree centrality*, or the number of ties in supporting spouse’s network), and quality of supporting spouse’s network will be positively related to the receiving spouse’s career outcome measured by the count of projects postmarriage.

Model 1 in Table 2 evaluates the impact of the control variables on our DV, the count of projects 3 years after marriage. Model 2 illustrates the significant impact that the supporting spouse’s degree centrality has on the receiving spouse’s postmarriage count of film projects. While we controlled for gender, age, industry experience in years before marriage (BM), awards before marriage, type of role, and change of role after marriage (AM), the relationship between Degree centrality and count of projects after marriage is significant (.0051, *p* < .01). This finding shows that both spouses benefit from marriage, and the added social capital and information flows confer on them in the form of an increase in movie roles. The finding confirms Burt’s (1998) work on borrowed social capital and Gorji et al.’s (2019) work on the value of family-generated social ties. Furthermore, it also confirms the importance of social network size and access to networks for information and employment opportunities in project-based environments such as Hollywood (Faulkner & Anderson, 1987; Jones, 1996; Jones & Walsh, 1997). In other words, the size of a spouse’s social network matters for getting cast in more projects.

**Table 2.** Binomial Regression: Count of Movies in the 3 Years After Marriage.

	Model 1	Model 2	Model 3	Model 4
Receiver’s gender	0.1454 (0.0961)	0.1653 (0.0961)	0.1653 (0.0960)	0.1967* (0.0969)
Receiver’s age at marriage	−0.0169** (0.0059)	−0.01499* (0.0058)	−0.0165** (0.0059)	−0.01629** (0.0060)
Receiver’s experience BM	−0.0573 (0.0657)	−0.0706 (0.0660)	−0.0602 (0.06565)	−0.05418 (0.0659)
Receiver’s count of project BM	0.4998** (0.0487)	0.48008** (0.0482)	0.4850** (0.0486)	0.44396** (0.0508)
Receiver’s type of role BM	−0.0077 (0.0817)	−0.0101 (0.08179)	−0.0114 (0.0818)	−0.0185 (0.0822)
Receiver’s award BM	0.7291** (0.2240)	0.7098** (0.2238)	0.7268** (0.2234)	0.7246** (0.2245)
Receiver’s change of role AM	0.1619 (0.0647)	0.1511 (0.0646)	0.1588 (0.0647)	0.1423* (0.0650)
Supporter’s degree		0.0051*** (0.0016)		
Supporter’s Eigen centrality			0.02094*** (0.0078)	
Supporter’s constraint				7.1656*** (1.167)
Constant	−0.443**	−0.567**	−0.599**	0.582**
Observations	1,168	1,168	1,168	1,168
χ^2^	123.96***	132.60***	130.70***	141.1***

*Note*. BM = before marriage. AM = after marriage. Standard errors in parenthesis. The values with a p-value less than 0.01 are indicated in boldface.

* *p* < .1. **p < .05. ***p < .01.

Hypothesis 1b states that the *quality* of the ties that exist in supporting a spouse’s network, that is, *Eigen centrality*, will be positively related to the receiving *spouse’s career outcome* postmarriage. As a proxy for the quality, we use two measures: Eigen centrality and Burt’s constraint. Models 3 and 4 in Table 2 illustrate the significant impact that the quality of supporter’s network has on the count of film projects in which the *receiving spouse* participates following marriage.

The statistical analysis shows that the relationship is significant for Model 3, supporter’s Eigen centrality (.0209, *p* < .01), a spouse’s high-status social network helps the receiving spouse. Model 4 shows that the effect of Burt’s Constraint is significant (7.165, *p* < .01) but not in the direction we expected. A positive constraint means greater network closure, or more social capital derives from a closed network with many redundant ties. However, our reasoning suggested a lower constraint would be associated with a supporting spouse’s capacity for bridging structural holes, which we expected would permit spousal referrals to other Hollywood people beyond a spouse’s closer network. Instead, unexpectedly, we find the opposite; network closure has a beneficial effect on postmarriage projects. The two results provide mixed support for Hypothesis 1b. Specifically, they contradict Burt’s (1992) theory about the value of structural holes in and between networks. Interestingly, these results show that more network closure in a supporting spouse’s network results in more valuable social capital to be lent to *a receiving spouse*. In the movie industry, a supportive spouse’s closed network appears to provide a useful asset that can benefit a receiving spouse, suggesting the existence of a closed “inner circle” or clique, which can be joined by spouses, a point to which we return in the discussion below.

Hypothesis 2 states that a supporting spouse’s social capital can provide greater access to higher status roles in the form of managerial positions than those available to a receiving spouse before marriage. To test Hypothesis 2, a multinomial logistic regression that tests changes in spouses’ postmarriage roles is used. Table 3 contains the results of the multinomial logistic regression.

**Table 3.** Multinomial Regression: Change of Roles After Marriage.

	Model 1	Model 2	Model 3
Change of roles: DEMOTE			
Receiver’s age at marriage	0.015* (0.007)	0.016* (0.007)	0.017* (0.007)
Type of marriage termination	0.290 (0.258)	0.392* (0.219)	0.306 (0.261)
Receiver’s award BM	−14.011 (11.849)	−13.968 (11.816)	−14.206 (11.954)
Receiver’s count of movies BM	−0.750 (0.536)	−0.978 (0.778)	−0.672 (0.446)
Receiver’s degree centrality BM	−0.050** (0.017)	−0.061** (0.021)	−0.050** (0.017)
Receiver’s Eigen centrality BM	−0.368 (0.256)	−0.271 (0.198)	−0.363 (0.221)
Receiver’s constraint BM	0.862 (0. 671)	0.834 (0.661)	0.823 (0.631)
Supporter’s degree centrality BM		−0.002 (0.001)	−0.003 (0.002)
Supporter’s Eigen centrality BM		−0.023 (0.003)	−0.049 (0.054)
Supporter’s constraint BM		−1.631 (1.697)	−0.919 (0.452)
Gender		−0.461** (0.165)	−0.597** (0.240)
Gender * Supporter’s degree centrality BM			0.091 (0.064)
Gender * Supporter’s Eigen centrality BM			0.063 (0.046)
Gender * Supporter’s constraint BM			7.553 (5.957)
Constant	−1.862** (0.345)	−1.8348** (0.356)	−1.643** (0.363)
Change of roles: PROMOTE			
Receiver’s age at marriage	0.040* (0.018)	0.031* (00.015)	0.041* (0.018)
Type of marriage termination	−0.465* (0.210)	−0.373* (0.181)	−0.489* (0.211)
Receiver’s award BM	0.334 (0.304)	0.412 (0.407)	0.495 (0.372)
Receiver’s count of movies BM	0.084 (0.061)	0.058 (0.046)	0.066 (0.051)
Receiver’s degree centrality BM	−0.005* (0.002)	−0.005* (0.002)	−0.005* (0.002)
Receiver’s Eigen centrality BM	0.028 (0.020)	0.081 (0.070)	0.061 (0.049)
Receiver’s constraint BM	0.046 (0.031)	0.082 (0.058)	0.079 (0.061)
Supporter’s degree centrality BM		−0.007 (0.006)	−0.003 (0.003)
Supporter’s Eigen centrality BM		−0.024 (0.019)	−0.049 (0.038)
Supporter’s constraint BM		−0.997 (1.171)	−0.919 (1.052)
Gender		0.216** (0.091)	0.193 (0.162)
Gender * Supporter’s degree centrality BM			0.003 (0.002)
Gender * Supporter’s Eigen centrality BM			−0.076 (0.045)
Gender * Supporter’s constraint BM			7.613 (4.724)
Constant	−0.289 (0.262)	−0.253 (0.220)	0.2125 (0.271)
Observations	1,168	1,168	1,168
χ^2^	145.58***	152.13***	165.18***

*Note*. BM = before marriage. AM = after marriage. Standard errors in parenthesis.

* *p* < .1. **p < .05. ***p < .01.

We coded change of roles as follows: Demote (1), No change (2), and Promote (3). Table 3 is divided into two main sections demote and promote. In Model 2, where we tested the effect of our three main measures of supporting spouse’s network variables on change of roles, we did not find any support for this hypothesis. Among our control variables, we find that age (0.016, *p* < .1) and size of a receiving spouse’s network (−0.061, *p* < .05) to be significant concerning demotion from managerial to lower level roles. A receiving spouse’s network size (degree centrality) has a negative correlation, which means individuals with a smaller network before marriage are more likely to be demoted postmarriage. Interestingly, the type of marriage termination, which is also used as a control variable, tends to have a significant role in promotion (0.392, *p* < .01) and demotion (−0.373, *p* < .1) postmarriage. This might arise due to instability in the marriage relationship (Amato & Hohmann-Marriott, 2007).

Gender also plays a significant part in role-change for both promote and demote postmarriage. Gender has a negative coefficient (−0.461, *p* < .05), showing that the probability of postmarriage male demotion is decreased, compared with women. However, neither spouses’ social network measures significantly impacted ascending or descending the career hierarchy. However, an upward change of role to managerial level, promote in our analysis, showed a significant positive coefficient for gender (0.216, *p* < .05). The significance of this gender result paves the way for our gendered bases Hypothesis 3, which predicts that women benefit more than men from borrowed social capital after marriage. We first performed a *t* test to determine whether there exists a significant difference between men and women regarding their project count before and after marriage. We split the sample according to gender to consider the performance effects before and after marriage through an independent *t* test. Table 4 shows the descriptive statistics of the test. As shown in Table 4, the average number of movies after marriage increases for both men and women.

**Table 4.** Group Descriptive Statistics: Count of Projects Before and After Marriage Between Genders.

	Gender	*N*	Mean	*SD*	*SEM*
Change of roles: 3 years after marriage	Male	584	1.43	0.911	0.054
	Female	584	1.35	0.857	0.048

Before marriage, men (average annual number of movies of 1.9) enjoy a higher count than women (annual average of 1.23), a mean difference of 0.68 (see Table 5). The average number of movies after marriage increases for both, but the increase in the male average (mean = 2.05) is higher than their spouse’s average (mean = 1.52). Table 5 presents an independent sample *t* test for comparing the performance of men and women before and after marriage using Levene’s test for equality of variance, *t* test for the equality of means. Both tests resulted in significant differences between the two groups before and after marriage, although we cannot attribute the increase to marriage.

**Table 5.** Independent Samples Test: Equality of Variances and Equality of Means—Count of Projects.

		Levene’s test for equality of variances		*t* Test for equality of means				
		*F*	Sig.	*t*	*df*	Sig. (2-tailed)	Mean difference	Standard error difference
3 Years before marriage count of projects	Equal variances assumed	30.783	0.000	5.81	1166	.000	0.68	0.116
	Equal variances not assumed			5.81	1096.76	.000	0.68	0.116
3 Years after marriage count of projects	Equal variances assumed	3.99	0.046	3.76	1166	.000	0.53	0.141
	Equal variances not assumed			3.76	1165.92	.000	0.53	0.141

Knowing that men and women differ regarding their performance measured by the number of projects, we continue our analysis to analyze possible gender differences in role change. Table 6 presents the group descriptive statistics concerning the change of roles. As mentioned before, promote is coded as 2, no change as 1, and demote as 0. If women do move to a higher status role after marriage, the mean of the change should be higher.

**Table 6.** Group Descriptive Statistics: Change of Roles After Marriage Between Genders.

	Gender	*N*	Mean	*SD*	*SEM*
3 Years before marriage count of projects	Male	584	1.9	2.217	0.092
	Female	584	1.23	1.715	0.071
3 Years after marriage count of projects	Male	584	2.05	2.408	0.119
	Female	584	1.52	2.427	0.102

However, as depicted in Table 6, the mean of change for women is 1.35 and is smaller than men’s 1.43. The difference between the groups is significant and is illustrated in Table 7 (*F* = 8.654, *p* < .01; *t* = −1.68, *p* < .1, and *t* = −1.67, *p* < .1, for equal variance and nonequal variance, respectively). This finding offers no support for Hypothesis 3, that women will benefit more from marriage. To complete our analysis for the difference between the genders, we also performed an interaction of gender with spouse’s measures of social capital in Model 3 of Table 3. Gender proved to be negative significant in demote in both Model 2 (−0.461, *p* < .05) and Model 3 (−0.597, *p* < .05). Indeed, when we consider Model 2 in Table 3, in the promote section, gender has a significant positive coefficient (0.216, *p* < .05), meaning that the probability of promotion to higher status roles will increase as we move from women to men. We found no support for the moderating effect of gender and the size of the spouse’s social capital.

**Table 7.** Independent Samples Test: Equality of Variances and Equality of Means—Change of Roles.

		Levene’s test for equality of variances		*t* Test for equality of means				
		*F*	Sig.	*t*	*df*	Sig. (2-tailed)	Mean difference	Standard error difference
Change of roles: 3 years after marriage	Equal variances assumed	8.654	.003	−1.68	599	.093	−0.121	0.072
	Equal variances not assumed			−1.67	580.56	.094	−0.121	0.072

The findings show that males are more likely to obtain managerial roles as producers or directors once married. The supporting spouse’s social capital has no significant impact on the change of roles and moving higher or lower in the hierarchy. This finding is contrary to our Hypothesis 2 that female spouses would move to managerial positions due to the social ties that they borrow from their male spouses. In this regard, this element of the analysis is contrary to Burt’s (1998) finding that female career advancement resulting from borrowed social capital, likely from a male sponsor. In a nutshell, the data indicate that females find more employment, but not necessarily more work in higher level management roles. This is not to say that there is no upward movement in female spouses’ career trajectories, but the slope of that trajectory has a modest incline (see Table 6—the average mean of role change comparison for men [1.43] vs. and women [1.35]).

## Discussion

We depict Hollywood celebrity couples as a hybrid form of a business family. These families in business do not typically own a firm but leverage their talent and celebrity through a family enterprise we label a “nexus of contracts” (Gedajlovic & Carney, 2010). As Hollywood is a prototype of a project-based industry that temporarily integrates diverse, specialized knowledge resources and expertise, the implications of this research reach beyond the movie industry. Many North American and European economies are increasingly project-based (Lundin et al., 2015). Many scholars often refer to the project-based organization as “the future of work” (Sundararajan, 2017), with some estimates suggesting that by 2027, 58% of American workers will have some project-based or freelance work experience (MBO Partners, 2017). Intraprofession marriage is flourishing creative industries such as culture, media, and entertainment, high-technology, and the professions (Bellow, 2004; DeFillippi, 2015; Hobday, 2000). In many of these industries, we anticipate that the business family as a nexus of contracts will become a prevalent and variegated organizational form reflecting industry-specific forms of valuable GNTAs.

While we have focused upon celebrity capital, which is generalizable to a variety of arts and cultural industries (Le Breton-Miller & Miller, 2015), other types of GNTA, including tacit knowledge and related forms of social capital, such as reputation and status (Gedajlovic & Carney 2010), which may form the basis of business family competitive advantage in other project industries. For example, reputation in the sense of an individual or organization “being known for something,” (Lange et al., 2011, p. 155) can underpin a business family brand and may differentiate married couples in technical and professional fields. Indeed, married couples may be an ideal setting for cultivating and leveraging such assets in single project industries. For example, after several years of drawing inspiration from Renaissance era artists, David and Elizabeth Emanuel operated a small custom-made couture business in London where they developed a reputation among “high society” figures. The Emanuals were propelled to prominence when they were selected to design Lady Diana Spencer’s wedding dress for her marriage to Prince Charles in 1981. Subsequently, the Emanuals leveraged their celebrity to established TV media careers and developed global couture businesses.

An important contribution of our article is to draw attention to the role of business family dynamics in single project industries and, by extension, the rising gig economy. Indeed, we believe that the unwavering focus on the traditional family firm is likely to generate diminishing returns for family business scholars. In contrast, exploring hybridized and adaptive forms of families in business in contemporary networked and project industries enlarges family business scholars’ horizons.

Based on the functioning of Hollywood, these industry and organizational trends indicate that social networks, and the associated inalienable asset such as human capital and reputation, will become increasingly relevant determinants of business family effectiveness in project-based industries. Accordingly, our findings have profound implications concerning the importance of social networks, particularly for women, and for issues of gender bias and homophily in business. In particular, understanding the social networks–career performance relationship is vital in industries where participants strive for excellence and equity. Merit and competence are often challenging to assess in these contexts (Le Breton-Miller & Miller, 2015). Indeed, the ambiguity surrounding project recruitment criteria for positions where individual excellence and equity are both desirable outcomes fosters heated debate. Specifically, we explore how the size and quality of a spouse’s network can instrumentally contribute to the professional careers of another individual. From this instrumental perspective, we can imagine intraprofessional marriage as a dynastic strategy, a form of reciprocal nepotism, or a type of sponsored recruitment and placement system specifically activated to realize mutual interests (Bellow, 2004). In the Hollywood production system, the cultivation and maintenance of social networks are imperatives, a necessary correlate of effective career management within the industry. It is from this perspective that we consider the article’s contributions. First, we consider our contribution to the family business literature. Second, we discuss some of the implications for the future of career progression in professions with project-based structures, and third, we also consider the persistence of gender inequity.

First, we call attention to the seemingly incongruous role of kinship and marriage as a mechanism for career advancement. We say *incongruous* since family-sponsored progression in a professional, knowledge-based industry is considered anachronistic. Instead, many people expect that career performance will reflect meritocratic criteria, such as educational attainment, talent, ability, and a range of social and psychological competencies. Preferment in rational-bureaucratic industries based on kinship through nepotism and unmerited favoritism is generally associated with mediocrity. Nevertheless, we find that spousal sponsorship is alive and flourishing in globally competitive network industries such as Hollywood.

We find much support for our Hypothesis 1a and some aspects of Hypothesis 1b. Specifically, we find a significant association between intraprofessional marriage and an improved number of film projects for both partners. Improved career performance in terms of film projects are associated with a function of the spouses: (a) network size, or degree centrality; (b) females though not males, benefit significantly from their spouse’s network status, or Eigen centrality; and (c) both partners benefit through the extent of their spouse’s association with a closed network closure or Burt’s constraint. Interestingly, a low Burt’s (1992) constraint or an open network with structural holes typically associated with finding the job (Granovetter, 2018). We did not anticipate this latter finding with our theorizing for Hypothesis 2. But the finding appears to result from an industry in which individuals become tightly connected within cliques. However, based upon the Transaction Cost Economics concepts of recurrent and relational contracting (Williamson, 1990) recurrent recruiting from cliques may facilitate stability and common reference points for coordinating individual project tasks in nonhierarchical structures (Narayan & Kadiyali, 2016; Starkey et al., 2000). Marriage provides an entrée into these tightly connected groups with an increased probability of being cast in a movie project. In other words, following intraprofessional marriage, both men and women benefit from the size and quality of their spouse’s social network. To clarify, we are not suggesting that Hollywood couples decide to marry to improve their career prospects. Rather, we suggest our findings represent a consequence of an epiphenomenon: There are many ways of cultivating career-enhancing social networks in Hollywood; marriage to a spouse with an extensive, high-quality network is one of them.

Marriage has long been an instrument of dynastic business family maintenance (Landes, 2008; Lisle-Williams, 1984). However, by drawing upon social class creation and reproduction theories (Bika & Fraser, 2020; Kuusela, 2018; Palmer & Barber, 2001) our theoretical contribution to the business family literature represents a case of inbound theorizing (Jaskiewicz et al., 2020). We consider established business families as social elites who will seek to reproduce both their economic and social status in the prevailing class structure (Kuusela, 2018). This may entail either promoting kin within a family firm or roles beyond the firm by inserting family members into elite echelons of professional and social life. However, the context of elite structures is dynamic to which dynastic business families must adapt (Bika & Fraser, 2020). The growth of project organization as a source of high-status employment is a natural extension of this dynamic. Viewed from this perspective, our depiction of intraprofessional marriage in Hollywood provides insight into how elites maintain favorable economic and social outcomes in competitive and dynamic industries (DiMaggio & Garip, 2012).

Moreover, succession to a role within the firm is not an option for the children of our sample of celebrity couples because the enterprise is simply a nexus of contracts. In a study of the celebrity couple David and Victoria Beckham, an English footballer and former *Spice Girls* pop singer, about their construction of a family brand for their multiple ventures, Parmentier (2011) suggests that the family prefers to shield their children from the glare of the media. Like many business families, the Beckhams find that younger members may wish to find employment or pursue entrepreneurial ventures beyond the family radius (Zellweger et al., 2011). Nevertheless, multigenerational show business families are ubiquitous in Hollywood (Gorji et al., 2019). For example, the extended Coppola–Schwartzman–Cage show business family is its fourth generation.^1^ The prevalence of multigenerational show business families in Hollywood indicates that these families may cultivate industry-specific competencies and find ways of inserting their children and spouses into the Hollywood production network. Describing the proliferation of multigenerational families in law medicine, sports, music, the military, and politics, Bellow (2004, p. 460) concludes:

We seem to be witnessing the formation of a series of professional enclaves dominated by networks of established families. These families don’t monopolise the field in the manner of mediaeval guilds but they do employ dynastic strategies used by previous elites and their offspring have advantages of access and opportunity that others don’t enjoy. . . . Dynastic methods of cast formation don’t have to be taught they simply happen.

Most of these business families do not own or manage family firms but collectively, they constitute a substantial part of the economy, and it is a phenomenon that is underresearched in the family business literature.

### Intraprofessional Marriage and Career Ascent

The value of social networks to improve individual economic outcomes and career ascent is well established (DiMaggio & Garip, 2012; Granovetter, 2018; Seibert et al., 2001). However, our study also reveals limits to intraprofession marriage as means of promoting career progression. Scholars seldom consider the effect of marriage as a source of social network formation within knowledge-based industries, particularly concerning spouses working in the same industry. This omission is surprising given the growth of intraprofession marriage. Accordingly, we contribute to the career management literature in the context of project-based industries. The implication is as we predicted in Hypotheses 1a and 1b, suggesting that dual-career couples will have a more significant advantage in finding regular project assignments in these settings. However, we find little support for Hypothesis 2 regarding the role of social networks in advancing an individual’s career up a networked industry hierarchy. Notably, our results suggest alternative interpretations about progressing up a career hierarchy and directing or producer roles.

In this regard, there are limits to what marriage can do. In Hypothesis 3, we find that, following marriage, males experience promotion in the career hierarchy into production management roles. Yet, if males enjoy upward mobility, it does not appear to have anything to do with the size and quality of their spouse’s social network. Moreover, we find that females enjoy no comparable social mobility into production management roles. Our data do not significantly support Hypothesis 3, predicting that spouses’ social networks will dramatically impact female career mobility.

We base our logic for Hypothesis 3 upon Burt’s 1998 theory about the gender of social capital. Burt suggests that females’ upward mobility in a corporate hierarchy benefits more from sponsorship or borrowed social capital. However, Burt’s (1998) conclusions are based upon a study conducted in a large U.S. electronics firm, a formal bureaucratic organization. We suspect such well-established corporations are prone to social and economic pressures to adopt career evaluation and promotion practices that are more favorable to women. Additionally, over the past 30 years, the number of women in managerial roles has increased as some organizations value executive diversity and female managerial qualities (Duehr & Bono, 2006). However, within the context of transient project organizations, establishing female-friendly evaluation and promotion practices does not appear to be based upon corporate procedure but on industry-wide normative cultures and long-established traditions. It seems that female-friendly norms and practices have yet to diffuse into such settings. Consequently, there are no apparent safeguards for females concerning blatant gender bias. Unfortunately, it appears that in Hollywood, the glass ceiling for women for managerial roles may have thickened. Thus, the consequences in other project organization industries are not promising based on the Hollywood precedent. Therefore, whether more equitable career advancement strategies become normative within project organization-based industries remains a subject for future research.

### The Persistence of Gender Inequity

Since business families often overlook women for succession roles in family businesses (Martinez Jimenez, 2009), it is worthwhile highlighting the specific problems they will confront in project-based industries. The issue of rampant gender inequities in the Hollywood movie industry has recently attracted much public attention engendering a social movement intent on remedying this long-standing injustice. However, in addition to the phenomena of homophily and women’s tendency to self-select into lower status social networks, the dominant logic of the blockbuster strategy in Hollywood plays into the persistence of gender inequities in subtle but remedial ways. As noted above, producers and other executives seek to reduce the risk associated with the blockbuster strategy. They consider women to be high-risk, which harms women’s ability to attain senior production management roles. However, that perception is based on the industry’s lore as there is little evidence to support the perception. Thus, Hollywood executives build their risk mitigation strategies on a false premise. Undoubtedly, academic research on the comparative performance of males and females concerning the financial performance of Hollywood productions warrants further investigation. More generally, we might reasonably expect to see a greater diversity of strategies with different risk profiles in other project-organization industries. It remains an open question whether women fare better in project-organization industries with more varied strategies. On a more positive note, Powell et al. (2002) show that stereotypes and negative attitudes toward outgroups, such as women, can change over time. Suppose Hollywood is a forerunner for the project-based economy, the extent to which stereotypes of women in the U.S. film industry are, or can be changed, these findings would be of potential benefit for many other sectors of the economy.

Our findings have practical implications for business families and their advisers, who help business families navigate their intergenerational succession planning. Insofar as the Hollywood movie industry reflects social network dynamics and other project-based industries, our findings offer insight for both marriage choices and, more important, the cultivation of social networks, which differ for males and females. We suggest that understandings of social networks and sponsorship as mechanisms for promoting family interests beyond a family firm can expand and enrich the field of family business by opening up fruitful new terrain for future research about how families practice sponsorship in business and the professions. Marriage is an important event in the development of business families but equally whether other family events, such as divorce or the arrival of children, has a positive or deleterious effects on each spouse’s career is an issue for future research. In the interests of maintaining the vitality of the field, we encourage family business scholars to go beyond traditional areas of concern and focus on how families promote family members’ interests beyond the family business. Family business scholars should hold a competitive advantage in this regard, and by building upon it they may expand and enrich the field of family business studies.

## Notes

1. First-generation soundtrack composer Carmine Coppola was the father of actress Italia Coppola, director Francis Ford Coppola, and actress Talia Shire. Talia Shire married music composer David Shire and later married Jack Schwartzman, parents of actor and producer Jason, and filmmaker Robert Schwartzman. Nicolas Cage is the nephew of Francis Ford Coppola, and father of actor Weston Cage Coppola. Literature professor August Coppola is the father of actors Christopher and Marc Marc Coppola, Marc married to actress Elizabeth Coppola (nee Seton).
